# Proteomic and Metabolomic Profiling of *Deinococcus radiodurans* Recovering After Exposure to Simulated Low Earth Orbit Vacuum Conditions

**DOI:** 10.3389/fmicb.2019.00909

**Published:** 2019-04-29

**Authors:** Emanuel Ott, Yuko Kawaguchi, Natalie Özgen, Akihiko Yamagishi, Elke Rabbow, Petra Rettberg, Wolfram Weckwerth, Tetyana Milojevic

**Affiliations:** ^1^Department of Biophysical Chemistry, University of Vienna, Vienna, Austria; ^2^Planetary Exploration Research Center (PERC), Chiba Institute of Technology (CIT), Chiba, Japan; ^3^Department of Life Science and Technology, Tokyo Institute of Technology, Nagatsuta, Yokohama, Japan; ^4^Department of Radiation Biology, Institute of Aerospace Medicine, German Aerospace Center, Cologne, Germany; ^5^Department of Ecogenomics and Systems Biology, University of Vienna, Vienna, Austria; ^6^Vienna Metabolomics Center (VIME), University of Vienna, Vienna, Austria

**Keywords:** *Deinococcus radiodurans*, high vacuum exposure, dehydration, proteomics, metabolomics, molecular stress response

## Abstract

The polyextremophile, gram-positive bacterium *Deinococcus radiodurans* can withstand harsh conditions of real and simulated outer space environment, e.g., UV and ionizing radiation. A long-term space exposure of *D. radiodurans* has been performed in Low Earth Orbit (LEO) in frames of the Tanpopo orbital mission aiming to investigate the possibility of interplanetary life transfer. Space vacuum (10**^-^**^4^–10**^-^**^7^ Pa) is a harmful factor, which induces dehydration and affects microbial integrity, severely damaging cellular components: lipids, carbohydrates, proteins, and nucleic acids. However, the molecular strategies by which microorganisms protect their integrity on molecular and cellular levels against vacuum damage are not yet understood. In a simulation experiment, we exposed dried *D. radiodurans* cells to vacuum (10**^-^**^4^–10**^-^**^7^ Pa), which resembles vacuum pressure present outside the International Space Station in LEO. After 90 days of high vacuum exposure, survival of *D. radiodurans* cells was 2.5-fold lower compared to control cells. To trigger molecular repair mechanisms, vacuum exposed cells of *D. radiodurans* were recovered in complex medium for 3 and 6 h. The combined approach of analyzing primary metabolites and proteins revealed important molecular activities during early recovery after vacuum exposure. In total, 1939 proteins covering 63% of *D. radiodurans* annotated protein sequences were detected. Proteases, tRNA ligases, reactive oxygen species (ROS) scavenging proteins, nucleic acid repair proteins, TCA cycle proteins, and *S*-layer proteins are highly abundant after vacuum exposure. The overall abundance of amino acids and TCA cycle intermediates is reduced during the recovery phase of *D. radiodurans* as they are needed as carbon source. Furthermore, vacuum exposure induces an upregulation of Type III histidine kinases, which trigger the expression of *S*-layer related proteins. Along with the highly abundant transcriptional regulator of FNR/CRP family, specific histidine kinases might be involved in the regulation of vacuum stress response. After repair processes are finished, *D. radiodurans* switches off the connected repair machinery and focuses on proliferation. Combined comparative analysis of alterations in the proteome and metabolome helps to identify molecular key players in the stress response of *D. radiodurans*, thus elucidating the mechanisms behind its extraordinary regenerative abilities and enabling this microorganism to withstand vacuum stress.

## Introduction

With future long-term space explorations in mind, understanding the molecular mechanisms of survival in outer space becomes increasingly important. The vacuum and radiation-filled outer space provides hostile conditions to any form of life. However, there are some organisms that developed survival strategies for extreme environments on Earth that may also be favorable for their viability in outer space, most prominently the desiccation-resistant spores of *Bacillus subtilis* (Horneck et al., 2012) or *tardigrades* (Jönsson et al., 2008) in their multi-resistant tun state. Desiccation by space vacuum exposure (pressure below 10^-4^ Pa) is one of the most harmful factors to microorganisms in outer space, leading to severe changes on a proteomic and genomic level (Cox, 1993). One of the primary targets of dehydration is the lipid bilayer which can undergo a conversion of bilayer sheets to spherical micelles, subsequently affecting cell membrane associated proteins like porins and membrane-bound cytochromes as well (Cox, 1993). Furthermore, Maillard reactions can lead to amino-carbonyl reactions, causing cross-linking of proteins to other proteins, sugars and nucleic acid components (Supplementary Figure S1) (Cox, 1993). The resulting polymerization of biomolecules can alter crucial cell functions by changing membrane permeability, impeding enzyme function (Horneck et al., 2010) and subsequently major biosynthesis as well as transport and repair pathways. Desiccation-induced disturbance of the mitochondrial electron transport chain in combination with the disruption of protein function by Maillard reactions lead to an intracellular build-up of ROS in *Zea mays* (Billi and Potts, 2002; França et al., 2007). The accumulation of ROS ultimately results in a destructive biochemical cascade, reinforcing lipid peroxidation, denaturation of proteins and nucleic acid damage with severe consequences on overall cell metabolism (Hansen et al., 2006; Garcia, 2011).

Upon dehydration, DNA is prone to experience double strand breaks (DBS), as detected in spores of *B. subtilis* and in the gram-positive bacterium *D. radiodurans* after simulated outer space vacuum (10**^-^**^6^ Pa) and real outer space vacuum treatment (Dose et al., 1992, 1995). This observation is supported by various subsequent studies with DNA repair deficient mutants that exhibited decreased survival during high vacuum conditions (Horneck et al., 1995; Munakata et al., 1997). Moreover, a transcriptomic analysis of *B. subtilis* spores subjected to 1.5 years of outer space and simulated Mars conditions conducted by Wayne et al. indicated a DNA response unique to vacuum desiccation as a single factor (Nicholson et al., 2012). In this study, spores of *B. subtilis* were exposed to outer space on aluminum coupon stack triplets and were subsequently compared with spores subjected to simulated Martian environment. Spores retrieved from the middle and lower and therefore UV-shielded layer of the space vacuum aluminum coupons exhibited an overall much stronger and broader DNA damage response compared to the samples exposed to the UV-shielded Martian environment (Nicholson et al., 2012). The only differing parameter in terms of space related stress between both conditions was the surface pressure subjected on spores (3 Pa simulated Martian atmosphere vs. 10^-4^ Pa), thus highlighting the importance of high vacuum as an environmental factor (Nicholson et al., 2012). Interestingly, the DNA damage response of spores after UV-shielded space exposure differed from the classic DNA damage response. It was lacking elevation of *lexA* expression, a master regulator of the classic DNA damage response in *B. subtilis* (Nicholson et al., 2012). This suggests that vacuum desiccation may trigger a DNA damage response unique to outer space related stress factors (Nicholson et al., 2012).

The mutagenicity of space vacuum was first reported in the Spacelab1 experiment in 1984 (Horneck et al., 1984). Spores of histidine deficient *B. subtilis* that were exposed to vacuum (1.2 × 10^-4^ Pa) showed a tenfold increase of histidine revertant mutants compared to samples kept on 1 atm (1.0 × 10^5^ Pa) (Horneck et al., 1984). Additionally, it was possible to show that vacuum increases the susceptibility of *B. subtilis* to ultraviolet radiation by a factor of 1,2 to 9,1 (Horneck et al., 1984). Further investigations of *B. subtilis* spores from strains subjected to high-vacuum (1 × 10^-3^ Pa) exposure by Munakata et al. (1997) indicate that mutation frequencies increase proportional to vacuum exposure time. In this study a 5′-CA to 5′TT tandem double base exchange located at codon 84 of the *gyrA* Gene was identified, which occurred in 55 to 62% of nalidixic acid-resistant mutant spores of the strains HA101 (*hisH101, metB101, leuA8*) and TKJ6312 (*uvrA10, spl-1*), respectively (Munakata et al., 1997). Strikingly, out of more than 500 mutants obtained after various treatments, this specific double base exchange mutation was reported to only arise in spores that were vacuum treated (Munakata et al., 1997).

In our study we focus on the vacuum-stress response of *D. radiodurans*, a gram-positive microorganism highly resistant against various extreme environmental conditions. In contrast to *B. subtilis* it does not resort to spore formation to sustain under extreme conditions (Dose et al., 1992). With its highly effective and fast DNA repair mechanism (Zahradka et al., 2006; Slade et al., 2009), in combination with its ROS-scavenging capacities by intracellular antioxidant complexes (manganese (Mn^2+^), orthophosphate (P_i_) and peptides) (Daly et al., 2004, 2007, 2010), it can withstand extended periods of time of extreme dryness (Mattimore and Battista, 1996; Fredrickson et al., 2008) and ionizing radiation with an acute dosage of 5,000 grays (Gy) with almost no loss in viability (Moseley and Mattingly, 1971; Ito et al., 1983). This not only qualifies *D. radiodurans* as a model organism to unravel the different molecular mechanisms for withstanding detrimental outer space conditions, it also makes it a compelling candidate for biotechnological advances under extreme conditions. Thus, *D. radiodurans* can be considered for future biotechnological applications such as bioremediation of radioactive waste on earth or as a biotechnical tool for future space exploration missions. Presently, multi-resistance of *D. radiodurans* has been exploited in a few successful biotechnological attempts. For instance, a recombinant *D. radiodurans* strain for bioremediation was engineered and shown to be effective (Appukuttan et al., 2006). This strain expresses the non-specific uranium precipitating acid phosphatase *phoN* (Appukuttan et al., 2006) and the mercury (Hg) (II) resistance gene *merA*, respectively (Brim et al., 2000). The latter gene encodes for an enzyme capable of reducing toxic Hg (II) into less toxic volatile elementary Hg (Brim et al., 2000). Gaining insight into the molecular basis of its multi-stress resiliency will further aid in the development of effective sterilization techniques for space ships to not only prevent disease outbreak but also unwanted contamination of extraterrestrial environments during space missions. In respect to its highly effective coping mechanisms with ROS induced stress, *D. radiodurans* can be used as a model to study the molecular mechanisms of cancer and aging (Slade and Radman, 2011). An experiment involving treatment of human cell lines with *D. radiodurans* ultrafiltrate indicated that the manganese complexes also aid other organisms against harmful radiation and ROS (Daly et al., 2010).

In ionizing-radiation-resistant bacteria, *S*-layer proteins might play an important role in response to radiation damage (Gentner and Mitchel, 1975). The *S*-layer, which is the first line of defense against environmental factors, appears to be extremely versatile. It is assumed that protein expression is adapted to different stress factors through rearrangements of DNA (Pollmann et al., 2006). These proteins are anchored to the cell surface via non-covalent interactions and are proposed to interact with the pink carotenoid deinoxanthin within *D. radiodurans* cell envelope (Ghedira et al., 2016). This interaction helps to protect *D. radiodurans* from UV radiation under desiccation conditions (Farci et al., 2016).

Overall, these characteristics make *D. radiodurans* an excellent candidate for studies involving outer space survival and interplanetary space travel. However, data of *D. radiodurans* susceptibility and molecular response to outer space parameters is sparse. In our study, we aim to investigate the molecular response of *D. radiodurans* to outer space vacuum as the sole factor. Therefore, we subjected *D. radiodurans* cells to 8.7 × 10^-5^ Pa (space simulating vacuum) for a duration of 90 days in the ground-based Astrobiology Space simulation facility at DLR Cologne, to simulate outer space vacuum conditions (Rabbow et al., 2016). Subsequent metabolomic and proteomic analyses as well as an inspection of RNA integrity were performed with dehydrated cells of *D. radiodurans* exposed to space-simulating vacuum. The experiment was conducted as a preliminary investigation prior to the Tanpopo mission, where *D. radiodurans* was kept under LEO conditions at the International Space Station (ISS) for a period of 1 to 3 years (Kawaguchi et al., 2016; Yamagishi et al., 2018). Herewith, we hope to contribute to studies based on outer space response of *D. radiodurans* (Pogoda de la Vega et al., 2007; Bauermeister et al., 2011), in order to provide an in depth understanding of the molecular response to vacuum as single factor.

## Materials and Methods

### Cultivation and Preparation of Dehydrated *D. radiodurans* Cells

Dehydrated *D. radiodurans* R1 cell layers with thickness of 1.4 mm were deposited in wells of a round aluminum plate as described previously (Ott et al., 2017). Briefly, *D. radiodurans* was cultured 15 h in TGB medium (1%(w/v) tryptone, 0.6%(w/v) beef extract, 0.2%(w/v) glucose) at 30°C in an incubator with shaking speed of 150 rpm until it reached the mid-exponential phase. Liquid cultures of *D. radiodurans* were washed in 10 mM phosphate buffer (PB): 10 mM K_2_HPO_4_, 10 mM KH_2_PO_4_, pH 7. This step was repeated three times. Aluminum plates containing cylindrical wells (2.0 mm diameter, 2 mm depth) with flat floor were used as sample holders (Kawaguchi et al., 2016). Twelve microliters of a cell suspension (2.9 × 10^9^ cells/mL) were dropped into 4 wells and dried up under 3.3 × 10^3^ Pa in a desiccator at room temperature (RT) under sterile conditions. These steps were repeated 6 times. The cells were dried up under 3.3 × 10^3^ atm for 16 h. Subsequently, cells were exposed to vacuum that mirror LEO conditions outside the ISS as close as possible (8.7 × 10^-5^ Pa) at 21°C for 90 days, whereas the controls remained in a desiccator at 21°C (Supplementary Figure S2).

### Recovery of Dehydrated *D. radiodurans* Cells

**FIGURE 1:**
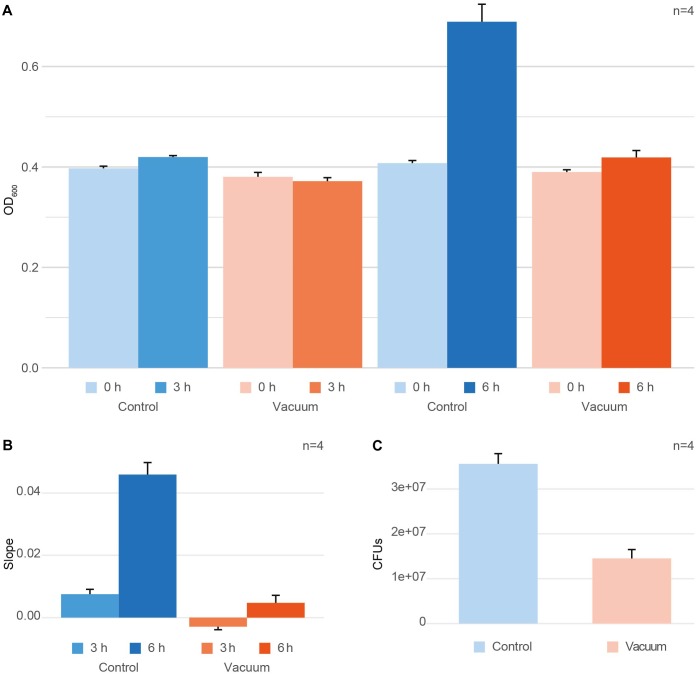
Effect of vacuum on survival and growth of *D. radiodurans*. **(A)** OD_600_ measurements at t_0_ (inoculation timepoint) and the harvesting timepoint for the control cells and vacuum exposed cells. **(B)** Changes in OD_600_ values between t_0_ and the harvesting timepoint per hour of cultivation shown as ratio per hour. **(C)** Colony forming units (CFUs), counted for the control cells and vacuum exposed cells. In case with CFU, samples for plating were picked at t_0_. Error bars always show the standard error based on the measurements of four replicates. Error bars at t_0_ represent the measurement error of the instrument.

After exposure to vacuum, cells were recovered from wells of aluminum plates using 10 mM PB followed by incubation with TGB medium at 80 rpm at 32°C. OD_600_ measurements were performed at zero time point t_0_ and the harvesting time points t_3h_ and t_6h_ (Figure 1A). To get a single, comparable value for each condition, the growth per hour between t_0_ and the harvesting timepoint was calculated (Figure 1B). Additionally, the growth of vacuum exposed and control cells was monitored using colony formation units (CFU) counting to evaluate the loss of cell viability induced by the vacuum exposure (Figure 1C). For CFU counts, t_0_ exposed and control cells were put on TGB agar plates in different dilutions and were incubated for 2 days at 30°C until colonies achieved a countable size.

### RNA Integrity

To determine suited timepoints for the extraction of metabolites and proteins, which allow insights in the early molecular response to the vacuum conditions, RNA integrity was evaluated. For RNA integrity measurements, two replicates for 3 h and two replicates for 6 h growth in a complex liquid medium were prepared (Supplementary Figures S3, S6).

To harvest the cells, the cultures were centrifuged (2000 *g*, 5 min, 4°C). The pellet was washed with 5 mL PB, 1.8 mL PB and 900 μL H_2_O with vortex and centrifuge steps (2000 *g*, 5 min, 4°C) in between. The pellet was stored at -20°C.

Silica beads and 1 mL TRIzol (Thermo Fisher Scientific) were added to the pellets and homogenization was performed with a FastPrepTM-24 Instrument (MP Biomedicals; 2^∗^60 s, 6.5 m/s). After bead beating, the mixture was incubated for 15 min at RT. 200 μL chloroform was added and samples were centrifuged (21000 *g*, 2 min, 4°C). The upper, polar phase containing RNA was transferred into a new tube.

To precipitate the RNA, 1,5 ml of 100% ethanol was added to the polar phase of each sample and incubated at RT for 10 min. The RNA supernatant containing ethanol was centrifuged (12000 *g*, 2 min, 4°C) using EconoSpin^®^ Silica-Membrane Mini Spin Columns. Membrane-bound RNA was washed with 750 μL 90% and subsequently 70% ethanol and centrifuged between each washing step (12000 *g*, 2 min, 4°C). The column was centrifuged afterwards with maximum speed for 1 min to evaporate ethanol residuals. The RNA was eluted using 50 μL RNAase free H_2_O (6000 *g*, 2 min, 4°C), after incubation for 1 min at RT. RNA integrity was evaluated by performing a 1% Agarose Gel Electrophoresis. Into each slot 1000 ng of RNA were loaded.

### Simultaneous Extraction of Proteins and Metabolites

For the integrative extraction of proteins and metabolites a modified protocol according to Weckwerth et al. (2004) was used (Valledor et al., 2014). The sample extraction procedure is illustrated in Supplementary Figure S4.

The content of 14 wells with the vacuum exposed cells and 14 wells with the control cells were resuspended in 15 mL PB each. The suspensions were used to inoculate eight 250 mL flasks containing 30 mL of TGB medium for exposed and the control conditions. Cultures were incubated at 80 rpm at 32°C for 3 h and 6 h, respectively. 4 replicates for the vacuum exposed and 4 replicates for the control cells were incubated for 3 h. Additionally, 4 replicates for the vacuum exposed and 4 replicates for the control cells were incubated for 6 h. Cells were harvested as described in the previous paragraph “RNA integrity” and homogenization settings remained the same. Instead of TRIzol, 1 mL of ice-cold MCW (methanol:chloroform:water; 2.5:1:0.5) was used as solvent. After homogenization, samples were incubated 15 min on ice and centrifuged (21000 *g*, 4 min, 4°C). The supernatant, which contained metabolites, was transferred into a new tube for the subsequent purification of primary metabolites. The pellet, containing proteins and nucleic acids, was washed with 1 mL methanol, centrifuged and air-dried (21000 *g*, 4 min, 4°C). TRIzol was added to the dried pellet and it was additionally homogenized in the bead beater (30 s, 6.5 m/s). After bead beating, the mixture was incubated for 15 min at RT. Chloroform (200 μL) was added and samples were centrifuged (21000 *g*, 2 min, 4°C). The lower, apolar phase was transferred into new tubes for protein purification. The apolar phase (∼550–600 μL) was washed once more with 550 μL H_2_O, centrifuged (21000 *g*, 2 min, 4°C) and transferred into new tubes. Finally, 1.5 mL 0.1 M NH_4_Ac in methanol with 0.5% beta-mercaptoethanol was added and proteins were precipitated over night at -20°C.

#### Shotgun Proteomics

##### Protein quantification and in-solution digestion

Protein pellets from the extraction step described above in 2.4 were centrifuged (21000 *g*, 15 min, 4°C), the supernatants were discarded, the pellets were washed two times with 1.8 mL ice-cold methanol and one time with 1.8 mL ice-cold acetone. For each washing step, the pellets were ultrasonicated for 5 min, centrifuged (21000 *g*, 15 min, 4°C) and the supernatants were discarded. After the final washing steps, pellets were air dried.

Pellets were resuspended in 40 μL 8.8 M urea in 50 mM HEPES on a shaker for 30 min at 750 rpm. After centrifugation (21000 *g*, 5 min, RT), a BCA (bicinchoninic acid assay) was performed to determine the protein concentration against different BSA concentrations (Supplementary Figure S5).

For digestion, 60 μg proteins of each sample were used. With the urea/HEPES buffer sample volumes were filled up to 15 μL. As a reduction step, samples were adjusted to 5 mM dithiothreitol (DTT) and incubated for 45 min on a thermoshaker at 37°C at 700 rpm. Afterwards, samples were alkylated by adjusting the iodoacetamide (IAA) concentration to 10 mM, followed by incubation for 60 min in dark on a thermoshaker at RT at 700 rpm. Alkylation was stopped by adjusting the DTT concentration to 10 mM DTT (total sample volume was 29.3 μL) and samples were further incubated for 15 min on a thermoshaker at RT. Before digestions, 29.3 μL 20% acetonitrile (ACN) 100 mM ammonium bicarbonate and 58.6 μL 10% ACN 25 mM ammonium bicarbonate and 10 mM CaCl_2_ were added to the samples. Three microliter of trypsin beads (Promega) were added to digest proteins. Samples were incubated at 37°C at 10 rpm for 16 h.

##### Desalting and peptide quantification

To stop digestion, samples were put on ice. To desalt samples a C18 spec plate (Agilent) connected to a water-jet pump was used. The C18 membrane was activated with 2 × 800 μL methanol and washed with 2 × 800 μL H_2_O without incubation time in between. Samples were acidified by adding 10 μL 20% formic acid, centrifuged (21000 *g*, 2 min, 4°C), loaded on the C18 material and incubated for 10 min at RT. Peptides were washed on the C18 material with 2 × 800 μL water and finally eluted with 3 × 200 μL methanol. Samples were dried down in a speedvac.

To determine digestion efficiency and to normalize the peptide amount throughout all samples, a colorimetric peptide quantification assay (Pierce) was performed (Supplementary Figure S5) after resuspending samples in 100 μL 2% ACN 0.1% formic acid. Samples were further diluted to a peptide concentration of 50 ng/μL.

##### HPLC nESI-MS/MS measurement and data analysis

For shotgun proteomics measurements, 5 μL of each sample were injected into an nHPLC-Orbitrap Elite (Thermo Fisher Scientific, Bremen, Germany), measurement settings were described before (Ott et al., 2017). Data analysis was performed with Maxquant (Cox and Mann, 2008). The minimum peptide length for identification was set to 7 amino acids and one unique peptide was required for protein identification (FDR 1%, based on target decoy database). For identification, measured spectra were compared to the *D. radiodurans* FASTA file from Uniprot (October 2018, 3085 sequences in the database). Further settings: 20 ppm first search peptide tolerance, 4.5 ppm main search peptide tolerance, maximum of 2 missed cleavages, maximum number of 5 modifications per peptide [variable: oxidation (M) and acetylation of protein *N*-term, fixed: carbamidomethylation (C)], label free quantification of samples. The mass spectrometry proteomics data have been deposited to the ProteomeXchange Consortium via the PRIDE (Vizcaino et al., 2016) partner repository with the dataset identifier PXD011868.

#### Derivatization and Analysis of Metabolites With GC-BT-TOF-MS

For metabolite measurements, 300 μL of H_2_O was added to the supernatants after cell homogenization to achieve a phase separation. Samples were centrifuged (21000 *g*, 2 min, RT), and the upper, polar phase was transferred into a new tube and 3 μL of 10 mM PGP (Phenyl β-D-glucopyranoside) was added as internal standard. Samples were carefully dried in a speedvac. Before measurement, methoximation and silylation with *N*-methyl-*N*-trimethylsilyltrifluoroacetamid to add trimethylsilyl (TMS) residues was performed as described elsewhere (Weckwerth et al., 2004). Measurement of polar metabolites was performed on a GC-BT-TOF-MS (Leco) instrument. Separation of metabolites was achieved on an Agilent 7890B gas chromatograph on a Restek Rxi-5 ms (30 m length, 0.25 mm diameter and 0.25 μm film) in split 10 mode with helium as the carrier gas. The following settings were applied: flow rate 1 mL min^-1^, injection temperature 230°C, column temperature start at 70°C for one minute, then heated up to 330°C in 9 min and hold for 8 min, ion source temperature at 250°C, acquisition rate 10 spectra s^-1^, recorded masses 50–600 m/z.

ChromaTOF (Leco) was used for peak integration. For our targeted approach, metabolites were identified based on a house-intern quality control mix, containing several primary metabolites of interest. For data processing, first areas <10000 were removed from the dataset, then every sample was normalized to the area of the internal standard. Areas of the blank (medium extract without cells) was subtracted from each measured substance and all derivatives of each metabolite were summed up. Finally, metabolites of every sample were normalized to the corresponding OD_600_ values.

The untargeted approach included a library search of all integrated peaks. Peaks with a similarity higher than 700‰ were annotated. As reference libraries, two GMD (Golm Metabolome Database) libraries and one NIST (National Institute of Standards and Technology) library were used. The hit with the highest similarity was chosen as annotation. Normalization was performed as described for the targeted approach.

### Statistical Evaluation of Data

Statistically, all data from proteomics and metabolomics measurements were treated the same. To avoid miscalculation of missing values, an ANOVA was only performed if the protein/metabolite was present in all replicates. However, the applied methods also allowed analyses of proteins and metabolites uniquely represented in a single sample. In case of metabolite data, the already normalized (to the OD-measurement) intensities were used, for proteins, LFQ (label free quantification) intensities, which were calculated by Maxquant (Cox and Mann, 2008) were used. Each metabolite and protein was *z*-scored over all samples. Subsequently, samples were separated in 4 groups (3 h control, 6 h control, 3 h vacuum and 6 h vacuum) and an ANOVA was performed to identify significant differences between these groups. To evaluate the reasons for the significance in the ANOVA, a *post hoc* test was performed. For both statistical tests the Perseus software (Tyanova et al., 2016) was used. All figures were created with the *z*-scored data (except fold change figures). Most figures were created in R (R Development Core Team, 2018) with corresponding packages: Boxplots, bar charts and scatter plot (ggplot2 Wickham, 2016), heatmaps (heatmap.plus Day, 2012), PCA (pca3d Weiner, 2017). The spider plot was created in Microsoft^®^ Excel and the TCA figure in Adobe Illustrator^®^.

## Results

### Effect of Vacuum on Survival of *D. radiodurans*

After exposure to vacuum conditions, dehydrated cells of *D. radiodurans* were recovered in complex medium and their survival was evaluated by CFU counts. Additionally, OD_600_ measurements of microbial cultures were performed with the recovering cells. The same measurements were applied towards non-exposed control dehydrated cells of *D. radiodurans*, which were stored in a desiccator at ambient temperature. Cells were harvested at 3 h and 6 h of recovery of the vacuum-exposed and control *D. radiodurans* cultures. At t_3h_, exposed cells showed a minimal decrease in OD_600_ values compared to t_0_, whereas the control cells revealed a marginal increase (not significant, Figure 1A). The OD_600_ values measured after 6 h of recovery in a complex medium indicated an active increase in culture density in control cells, while the cell density of vacuum-exposed cells was only slightly affected (Figure 1A). The control non-exposed cells showed a small increase of OD_600_ values after 3 h and a much higher increase after 6 h, while the vacuum-influenced cells displayed a delay in the increase of OD_600_ values. The change in OD_600_ per hour (slope) is significantly higher for t_6h_ control samples compared to all other conditions (Figure 1B). Additionally, CFU-counts for vacuum-exposed cells showed a 2.5-fold lower survival rate compared to the control non-exposed cells (Figure 1C).

### Metabolic Response to Vacuum

**FIGURE 2:**
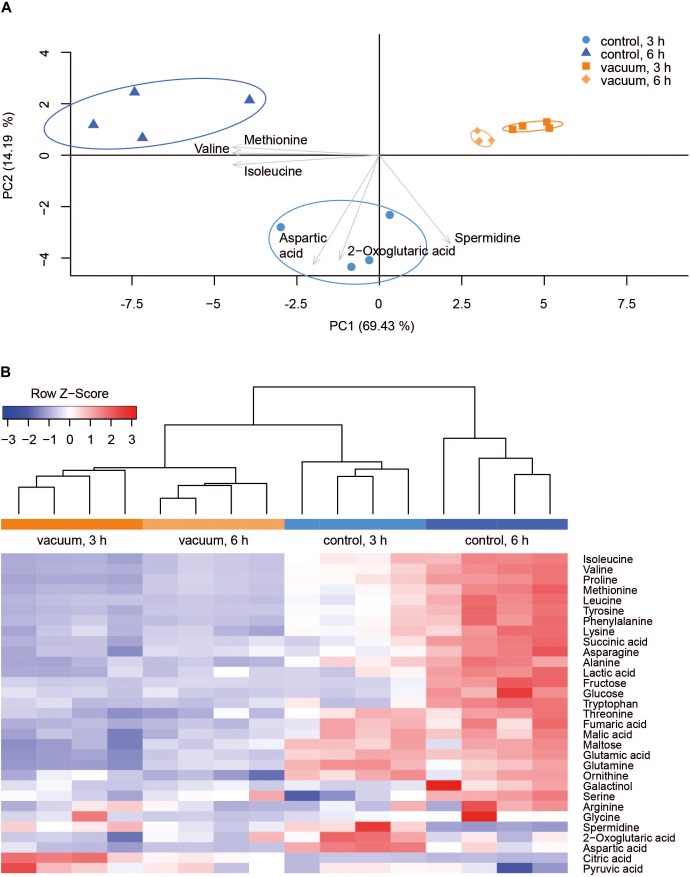
Metabolic response of *D. radiodurans* to vacuum (targeted approach). **(A)** Principal component analysis of 32 metabolites. The plot was created in R with the pca3d package. All data was *z*-scored, and the most impactful loadings are indicated as arrows. **(B)** Heatmap of measured metabolites with a corresponding dendrogram created in R with the heatmap.2 package. Metabolite data was *z*-scored before plotting and the dendrogram was drawn by the hclust function.

**FIGURE 3:**
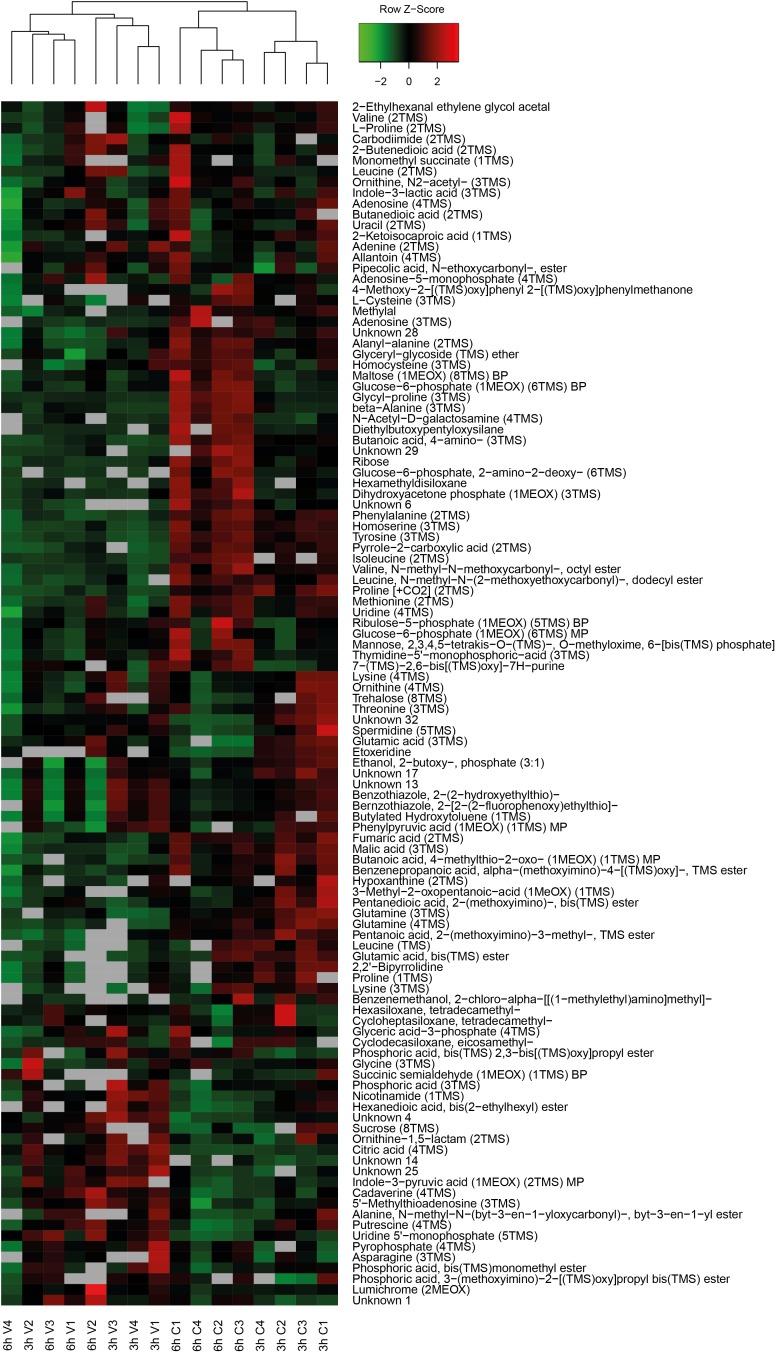
Heatmap of untargeted metabolites with the corresponding dendrogram. Data was *z*-scored before plotting.

Primary metabolites were measured in all four replicates after 3 h and 6 h of recovery of vacuum exposed and control cells. Results of the targeted analysis are presented in Figure 2 and Supplementary Table S1. On the Principal Component Analysis (PCA) (Figure 2A), all four data sets are clearly separated at the PC1 level, which explains approximately 70% of the variance. A general tendency is that control cells show much larger variation between 3 and 6 h of recovery than vacuum treated cells. Cells, which recovered for 3 h after vacuum exposure and control cells after 6 h of cultivation in a complex medium, were most different. According to the heatmap (Figure 2B), most amino acids are less present in vacuum exposed cells, especially after 6 h of recovery. In case of the untargeted approach, 252 peaks were successfully annotated. The number was reduced to 112, as only annotations present in at least 70% of the samples were used for further analyses (Figure 3 and Supplementary Table S2).

Most TCA cycle intermediates show an identical pattern (Figure 2B), although 2-oxoglutaric acid appears most abundant in control cells after 3 h of recovery. Two other exceptions are citric acid and pyruvic acid, being most abundant in vacuum exposed cells after 3 h of recovery. The slightly higher abundances of pyruvic acid and citric acid after vacuum exposure indicate that the input to the TCA cycle is very similar in control cells and in vacuum exposed samples. The main difference is the conversion rate of intermediates and products.

Spermidine is more represented after 3 h compared to 6 h in control as well as in vacuum exposed cells (Figure 2). Furthermore, the spermidine content from control cells was 2.7-fold reduced between 3 h and 6 h, whereas cells exposed to the damaging high vacuum showed only 1.3-fold reduction (Supplementary Table S1). In our untargeted approach, we were able to identify cadaverine (4TMS) and putrescine (4TMS) which were most present in vacuum exposed samples at t_3h_ of recovery. Other interesting candidates are nicotinamide (1TMS), indole 3 pyruvic acid, lumichrome and some not yet identified metabolites. Unknown 14 shows m/z values characteristic for carbohydrates and Unknown 25 is connected to carboxylic acids (Figure 3).

### Shotgun Proteomics Analysis

In total, 1939 proteins from *D. radiodurans* were identified in at least one sample, which represents a coding sequence coverage of approximately 63%. Out of these, 1166 proteins were quantified in each replicate and subsequently used for statistical analysis. The ANOVA (*p*-value ≤ 0.05) revealed 375 proteins as significantly different between the data sets. Differences between proteins from the vacuum exposed and control cells at both timepoints were determined by a *post hoc* test. After 3 h, 15 proteins were more abundant in the vacuum exposed compared to the control cells; 86 proteins were less abundant. After 6 h, 107 proteins were more abundant in the vacuum exposed cells compared to the control cells; 105 proteins were less abundant.

**FIGURE 4:**
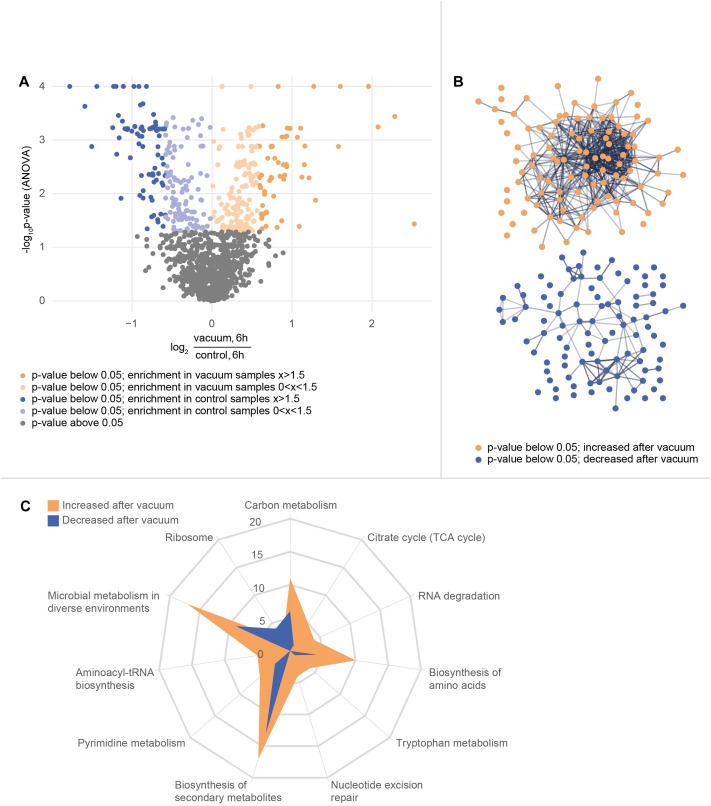
Proteomics analysis of the vacuum exposed and control cells after 6 h of recovery in a complex medium. **(A)** Volcano plot of all 1166 proteins that were identified in every replicate of every condition and timepoint. The *y*-axis plots the negative log_10_ corrected *p*-value (*q*-value) of the ANOVA. The *x*-axis shows the log_2_ fold change. All proteins with a fold change below 1.5 are indicated in brighter colors. **(B)** STRING database analysis from selected proteins. Proteins with a corrected *p*-value below 0.05 were divided into two groups. A *post hoc* test confirmed if there is a difference at the 6 h timepoint between control and vacuum exposed cells. 107 proteins were identified as higher abundant in vacuum exposed cells (orange group) and 105 proteins as less abundant (blue group). The STRING database was able to map 104 of the higher abundant ones and 98 of the lower abundant ones. Nodes are uploaded proteins and edges are interactions between proteins. The null hypothesis tests if the number of interactions could be assigned to any random set of proteins. The p-value for the orange set is 6.0^∗^10^-7^, the one for the blue set is 0.26. **(C)** KEGG pathway annotations were added to the uploaded proteins. The number of proteins from both sets of proteins which belong to several KEGG pathways is shown in the spider plot.

After 6 h of recovery we observed a similar amount of proteins that were more abundant in the vacuum treated cells compared to proteins which were less abundant (Figure 4A). Proteins with a *p*-value below 0.05 were divided into two groups and uploaded to the String database. The String database maps protein-protein interactions, connects interacting proteins with nodes and calculate the enrichment of those nodes (Szklarczyk et al., 2015). For proteins, which were less abundant after the vacuum exposure, no significant enrichment of nodes (*p*-value 0.260) was detected, whereas proteins, which were higher abundant after the vacuum exposure showed a high enrichment of nodes (*p*-value 6.02^∗^10^-7^) (Figure 4B). According to the String database, proteins which were higher abundant after 6 h of recovery in a complex medium, highly interact with each other. These proteins may work together to alleviate cell damage caused by the vacuum treatment. Proteins which were less abundant do not show a significant number of interactions. An enrichment analysis of proteins which were higher abundant identified several overrepresented KEGG pathways (Kanehisa and Goto, 2000). Proteins belonging to groups such as citrate cycle, nucleotide excision repair, aminoacyl-tRNA biosynthesis, microbial metabolism in diverse environments (Figure 4C) were more abundant after the exposure. Ribosomal proteins did not show a significant increase or decrease.

**FIGURE 5:**
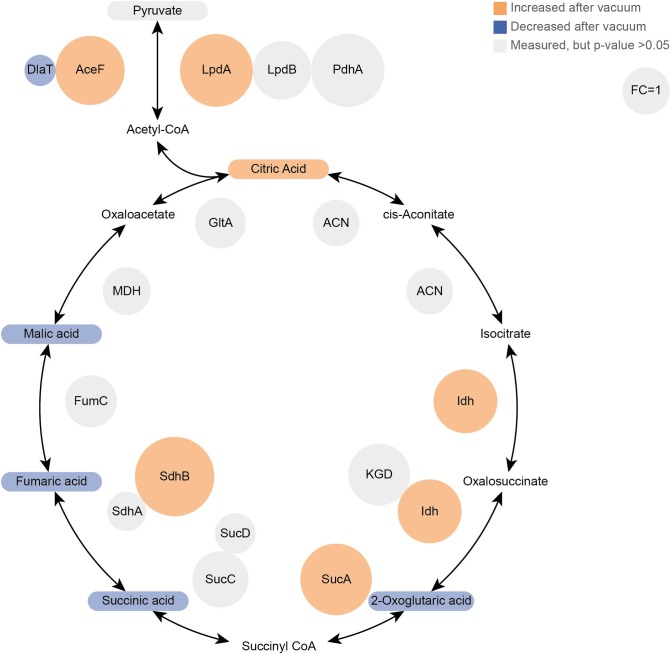
Proteomics and Metabolomics comparison of the TCA cycle between vacuum exposed and the control cells after 6 h of recovery in a complex medium. Metabolites are rounded rectangles and proteins are circles. Molecules in orange were more abundant after the vacuum exposure (ANOVA and *post hoc* test), blue molecules were less abundant. Gray ones were measured, but no statistical difference was calculated. In addition, the sizes of protein circles mirror their fold changes (vacuum 6 h/control 6 h).

**FIGURE 6:**
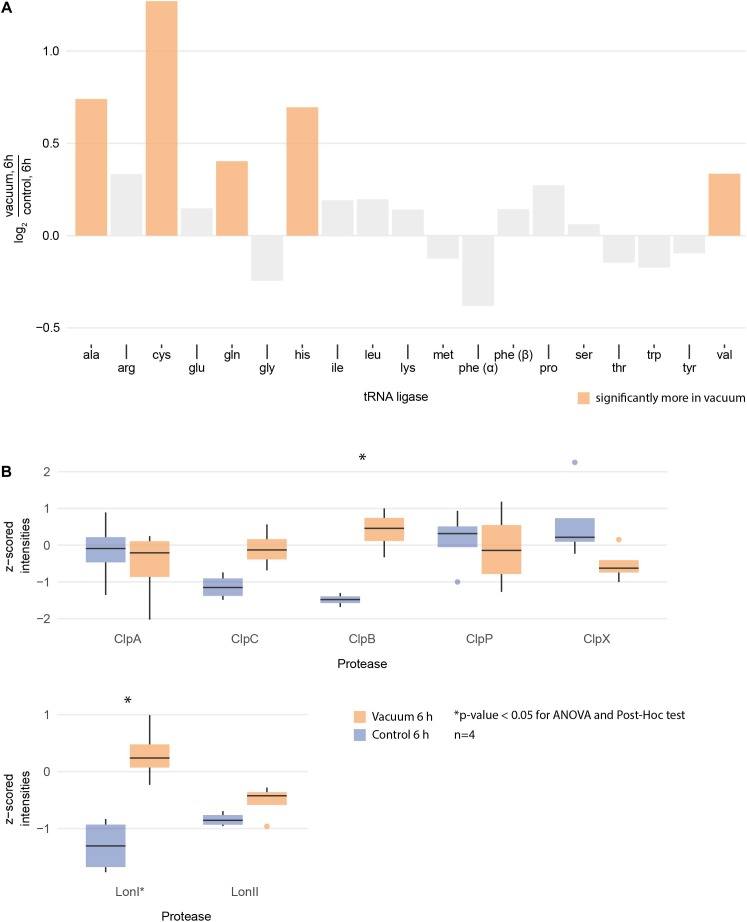
**(A)** Logarithmic fold change of all measured tRNA ligases between vacuum exposed and the control cells after 6 h of recovery in complex medium. The ones with statistically significant differences (ANOVA and *post hoc* test) are high lightened in color. **(B)** Normalized intensities of measured Clp and Lon proteases after 6 h of recovery between the vacuum exposed and the control cells. Statistically significant differences are indicated with a^∗^.

**FIGURE 7:**
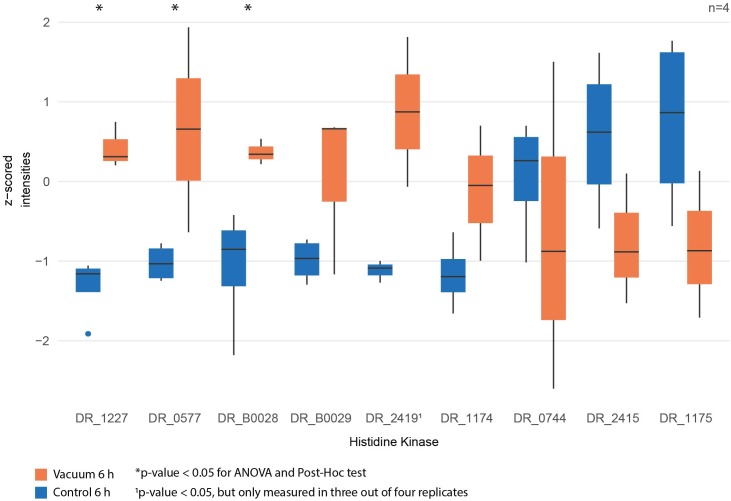
Normalized intensities of measured histidine kinases between vacuum exposed and control cells after 6 h of recovery in a complex medium. Statistically significant differences are indicated with a^∗^.

Proteomics analysis showed that after 6 h of cultivation in complex medium a lot of TCA cycle enzymes are more abundant in vacuum exposed cells (Figure 5). Furthermore, we observed an increase of some proteases (Figure 6B) during the recovery phase after vacuum treatment. Many t-RNA ligases (Figure 6A) that produce aminoacyl-tRNA (aa-tRNAs) were higher abundant at 6 h of recovery of vacuum exposed cells. These aa-RNAs are usually used by the ribosome for protein synthesis. However, as shown in Figure 4C, the enrichment analysis did not reveal a high abundance for ribosomal proteins after the vacuum exposure. It can be assumed that the non-proliferating, vacuum exposed cells after 6 h of recovery do not synthesize a lot of proteins, but still produce high amounts of aa-RNAs. An increase in proteins such as peroxidase DR_A0145, catalase KatA, several proteins involved in the UvrABC nucleotide excision repair machinery and polymerase PolA was observed during the first hours of recovery (Supplementary Table S3). To initiate all defense lines, intercellular signal cascades are undoubtedly important for a fast and efficient regulation of stress response. With our proteomics approach, we measured the abundances of several histidine kinases (Figure 7) at t_6h_ from vacuum exposed and control cells and identified several histidine kinases that were higher abundant in the vacuum exposed cells.

## Discussion

*Deinococcus radiodurans* is well known for its extraordinary resistance to radiation and desiccation. It was identified that ionizing radiation sensitive mutants are more vulnerable to desiccation (Mattimore and Battista, 1996), indicating that these effects trigger similar stress response mechanisms in the cell. Previously, it was shown that *D. radiodurans* can handle desiccation exceptionally well, but the exposure to high vacuum tremendously decreased survival of cells (Saffary et al., 2002). Our study supports these findings, as the number of colony forming units is reduced 2.5-fold (Figure 1C) after exposure of dehydrated *D. radiodurans* cells to high vacuum compared to dehydrated non-exposed control cells. Vacuum-induced desiccation causes severe dehydration, promoting Maillard reactions of carbohydrates, proteins and nucleic acids which result in cross linking and errors in polymerization (Supplementary Figure S1). These give rise to functional changes, such as altered enzyme activity, changes in membrane permeability, and alteration of genetic information (Horneck et al., 2010). Survivors of dehydration stress which are recovered in a cultivation medium undergo a prolonged lag phase (Bucker et al., 1972). Figure 1A shows that at 6 h of recovery, OD_600_ values of the control cells nearly doubled. Cells exposed to high vacuum remained in a growth arrest phase for a longer period and therefore, the OD_600_ did not increase noticeably after 6 h. Apparently, during the growth arrest phase, cells are mostly engaged in repairing the damage caused by vacuum.

**FIGURE 8:**
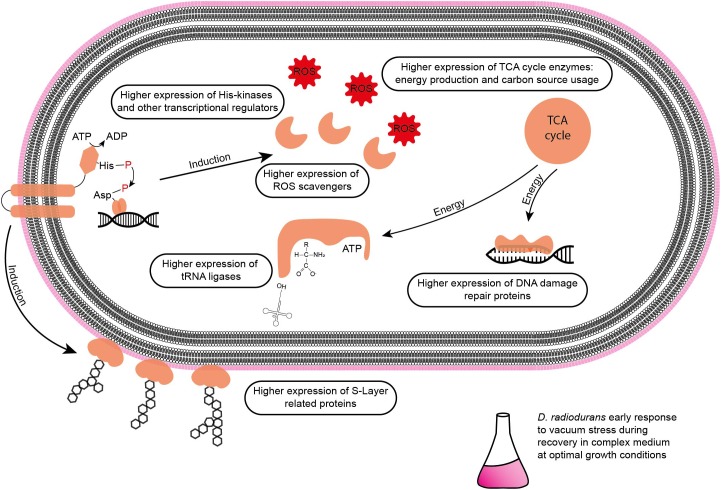
Summary of affected molecular components in *D. radiodurans* during the early stage of recovery from vacuum stress in a complex medium.

Although *D. radiodurans* can shield proteins from ROS induced damage (Daly et al., 2010), the amount of double strand breaks (DSB) is similar in all prokaryotic cells (Krisko and Radman, 2010). In addition to DSB, desiccation generates single strand breaks and base damage (Slade and Radman, 2011). Several repair pathways, e.g., base and nucleotide excision repair, mismatch repair and recombinational repair are used to fix damaged nucleic acids. Therefore, many proteins involved in these pathways appear least abundant in control cells at t_6h_ of recovery. This includes proteins involved in the UvrABC endonuclease repair (UvrA, UvrB, and UvrC), the endonuclease MutS2, the polymerase PolA and the gyrases GyrA and GyrB (topoisomerase). In previous studies, GyrA was identified to carry the majority of mutations induced by high and low vacuum in *B. subtilis* spores (Munakata et al., 1997; del Carmen Huesca Espitia et al., 2002). The higher abundance during the early phase of recovery indicates that various nucleic acid repair processes are ongoing. In *D. radiodurans*, RecA dependent DNA damage response is regulated by the transcriptional repressors LexA and LexA2 (DR_A0074). At t_3h_ we observed a higher abundance in LexA2 in vacuum exposed samples compared to control samples. However, RecA levels were lower in vacuum exposed samples. In a study with *lexA2* deficient mutants of *D. radiodurans* an increased amount of RecA was observed (Satoh et al., 2006). Therefore, we conclude that an increased level of LexA2 results in a low abundance of RecA, which delays DNA repair. The nucleic acid repair system in *D. radiodurans* is extremely efficient and our proteomics data shows an increased abundance of many repair related proteins in the early stages of recovery (Figure 8). However, there is no documented evidence that proteins typically involved in DSB repair appear higher abundant or with a higher specific activity in *D. radiodurans* compared to *E. coli* (Daly, 2009), although *E. coli* is about 30 times more susceptible to DSB than *D. radiodurans* (Slade and Radman, 2011).

### Role of Primary Metabolites and Energy Modulation After Vacuum Stress

Polyamines like spermidine and putrescine were postulated to be exploited by organisms from bacteria to plants and animals as a primordial form of stress molecules (Rhee et al., 2007). The exposure to oxidative stress induces polyamine synthesis, which leads to an expression of genes involved in ROS scavenging and repairing damage. In *E. coli*, transcription of catalases and other oxidative stress response proteins are induced by transcriptional regulons like RpoS and OxyR. The expressions of *rpoS* (starvation response) and *oxyR* (ROS response) are induced by polyamines in *E. coli* (Jung and Kim, 2003). Dehydrated cells exposed to high vacuum conditions and the control dehydrated cells showed an increase in spermidine after 3 h of recovery in a complex medium. The proteomics analysis revealed carboxynorspermidine decarboxylase, an enzyme that catalyzes the formation of spermidine from carboxyspermidine as significantly more abundant in t_3h_ compared to t_6h_. This indicates that polyamines are used as a general stress response during recovery from vacuum- and dehydration-induced stress.

Nicotinamide, a precursor of nicotinamide-adenine-dinucleotide (NAD), showed an increase in the early recovery phase (t_3h_) after vacuum exposure compared to the other conditions. NAD can be used as coenzyme for redox reactions and as substrate for NAD consuming enzymes, for instance ADP-ribose transferases (Gazzaniga et al., 2009). Lumichrome, a derivative of riboflavin, which is responsible for various extracellular processes in bacteria, such as quorum sensing signaling and extracellular electron transfer (Rajamani et al., 2008). In addition, riboflavin serves as precursor for flavin adenine dinucleotide (FAD), which is involved in redox reactions (Moreno-Hagelsieb et al., 2015). These two metabolites may contribute to oxidative stress response mechanisms in *D. radiodurans* after exposure to vacuum (Figure 8).

Cells, which were exposed to vacuum are metabolically less diverse during the growth arrest phase. We assume that in vacuum exposed cells, TCA cycle intermediates (2-oxoglutaric acid, fumaric acid, succinic acid, malic acid) and amino acids (Figure 2B, 5) are more rapidly enzymatically converted to support repair processes and therefore appear less abundant. In control cells, however, primary metabolites are not directly used, but rather produced as intended by the cells at logarithmic phase in optimal growth conditions. It can be concluded that vacuum treatment leads to an increase of TCA cycle intermediate conversion (Figure 8).

After exposure to desiccation stress many organisms showed an induction of proteins involved in the TCA cycle to produce the necessary amount of energy to alleviate cell stress (Riedel and Lehner, 2007; Gruzdev et al., 2012; Kocharunchitt et al., 2012). TCA cycle intermediates serve as precursors for amino acids, which are the preferred carbon source for *D. radiodurans* (Venkateswaran et al., 2000). Lon and Clp proteases presumably degrade damaged proteins to deliver more amino acids (Servant et al., 2007). However, the amount of amino acids after vacuum exposure is lower compared to control cells. We therefore looked for the correlation between the observed elevation in proteases and the decrease in amino acid abundance in recovering cells after the vacuum exposure. Several studies uncovered roles of aa-tRNAs as substrates in biochemical processes apart from protein synthesis (Raina and Ibba, 2014). Firstly, aa-RNAs can attach amino acids to the amino-terminus of damaged proteins as recognition sites for proteases (Mogk et al., 2007; Raina and Ibba, 2014). Furthermore, in *Streptomyces viridifaciens*, the antibiotic valanimycin is produced by transferring the seryl residue from seryl-tRNA to the hydroxyl group of isobutylhydroxylamine (Garg et al., 2006; Banerjee et al., 2010). Moreover, aminoacyl-tRNAs were shown to be involved in the formation of peptidoglycans as structural components of cell walls and membrane phospholipid modification (Shepherd and Ibba, 2013). Thus, we propose that the cell wall of *D. radiodurans* is one of the primary targets of vacuum-induced stress and that as a result, aa-tRNAs are recruited in order to recycle amino acids from the TCA cycle to aid in the reconstruction of damaged cell wall and membrane components (Figure 8).

### Regulation of the Vacuum-Induced Stress Response

Desiccation and high vacuum put cells under very stressful conditions, but even over a period of 90 days, *D. radiodurans* can survive and proliferate again, if the appropriate cultivation conditions are provided. Nevertheless, a growth arrest phase is necessary before proliferation of the vacuum exposed cells can be initiated. Our data indicates that ROS are eliminated, and macromolecules are repaired during this phase (Figure 8). Defective two-component signal transduction systems like histidine kinases and response regulators can increase susceptibility of *D. radiodurans* to various stress factors. In a knockdown study it was shown that some mutants that lack specific histidine kinases are less resistant to extreme conditions like ionizing radiation and UV radiation (Im et al., 2013). Our statistical analysis revealed that DR_1227 and DR_0577 were more abundant at 6 h after the vacuum exposure compared to the control cells at the same time point (Figure 7). These proteins belong to the less known type III histidine kinases (Kim and Forst, 2001) and might play an important role in the response to vacuum stress in *D. radiodurans*. Type III histidine kinases are usually part of chemotaxis signal transduction systems, but also appear in genomes that completely lack chemotaxis genes (Adebali et al., 2017). These histidine kinases always appear together with a putative marker gene for bacterial type IV pilus-based twitching motility (Kennan et al., 2015) (DR_0774), which might be regulated by them. In *D. radiodurans* this protein was identified as important part of the *S*-layer in the cell wall (Farci et al., 2014) alongside SlpA (DR_2577) (Farci et al., 2016). The role of these histidine kinases in the regulation of vacuum stress response needs to be further thoroughly elucidated.

To identify regulatory proteins that might be of special importance to vacuum response exclusively, only those which were higher expressed after 3 h and 6 h of recovery of the vacuum exposed cells were considered (Table 1). Out of these proteins, the histidine kinase DR_B0028, the type IV piliation system protein DR_0774, and the FNR/CRP transcriptional regulator DR_0997 are candidates that could contribute to the regulation of vacuum stress response. The histidine kinases DR_B0028 and DR_B0029 are thought to be co-regulated with an operon that encodes an antisigma factor-regulation system which is known to be involved in stress response in other bacteria (Hecker and Volker, 1998; Makarova et al., 2001). The aforementioned DR_0774 is a component of the *S*-layer in the cell wall. The *S*-layer coating on the external side of the cell wall together with the carotenoid deinoxanthin are involved in cellular protection from extreme environmental conditions, especially UV radiation after desiccation (Farci et al., 2016).

**Table 1:** Proteins which are more abundant in the vacuum exposed cells of *D. radiodurans* at 3 h and 6 h of recovery in a complex medium.

			The average *z*-scored intensities			
			
Protein IDs	Protein Annotation	ANOVA (*q*-value)	3h C	6h C	3h V	6h V
Q9RRY8	Uncharacterized protein	0.0004	-0.48	-1.23	1.13	0.58
Q9RVA2	Uncharacterized protein	0.0006	0.03	-1.36	1.11	0.23
Q9RY64	50S ribosomal protein L21	0.0013	-0.28	-1.22	1.06	0.44
Q9RXP1	Uncharacterized protein DR_0269	0.0006	0.09	-1.44	1.00	0.34
Q9RW95	Probable type IV piliation system protein	0.0006	-0.41	-1.22	0.97	0.66
Q9RVN0	Transcriptional regulator, FNR/CRP family	0.0001	0.32	-1.54	0.96	0.26
Q9RZS7	Uncharacterized protein	0.0077	-0.81	-0.75	0.88	0.69
Q9RZT5	Sensor histidine kinase/response regulator	0.0045	-0.48	-1.08	0.81	0.75


*The table shows Uniprot IDs, the corresponding protein annotations, the corrected *p*-values from the ANOVA and the average *z*-scored intensities for control (C) and vacuum (V) conditions. A color gradient runs from the lowest (saturated blue) to the highest (saturated red) value.*

FNR/CRP transcriptional regulators respond to a broad spectrum of intracellular and exogenous signals such as cAMP, anoxia, redox state, oxidative and nitrosative stress, 2-oxoglutarate, temperature (Körner et al., 2003). Apart from that, one of their family members, the DR_0997 protein also responds to high vacuum, according to our data (Table 1).

The results provided in this study are based on chromatographic separations coupled to mass spectrometers. These methods can be very powerful if misinterpretation is avoided. Bottom up/shotgun proteomics provides the possibility to relatively quantify several proteins from one organism, which is similar to combining multiple western blots. However, low abundant proteins might be under the limit of detection and as a result are not recognized by the detector (Zhang et al., 2013; Takáč and Šamaj, 2015). Furthermore, although the abundance is measured, the activity of a certain protein must be verified via enzymatic assays. GC-MS metabolomics based on reference substances avoids false positive identifications and allows exact relative and absolute quantification, while untargeted approaches combined with library searches offer many more identifications. In general, these studies provide important initial insights after applying a certain environmental stress to an organism but should be later investigated by targeted application of genetic and cell biological methods.

## Conclusion

It is generally accepted that vacuum induced dehydration of cells leads to Maillard reactions, which support the formation of ROS. Consequently, macromolecules are severely altered. Furthermore, the cell wall is affected, as metabolite transport through the membrane can be disrupted. In addition, the *S*-layer of cells can be damaged. Our study shows that high vacuum stress induces a prolonged growth arrest phase in *D. radiodurans* (Figure 1). This condition of suppressed growth is mirrored in our molecular analysis. The comparison of vacuum treated cells after 3 h of recovery towards corresponding control cells showed only minor variations on a molecular scale, as samples are still in growth arrest phase. However, at 6 h of recovery after the vacuum exposure *D. radiodurans* cells portray a completely different picture. Overall, at this time point we observed important differences between vacuum exposed and control cells (Figure 4–8). Combining these results, it is possible to decipher molecular key components, which are necessary for an efficient repair after the vacuum exposure (Figure 8). In all conditions (except the control cells at 6 h of recovery, which are already in the proliferation phase), higher abundances of ROS scavenging proteins, e.g., peroxidases and catalases, were observed (Figure 8 and Supplementary Table S1). Moreover, the amounts of nucleic acid damage repair proteins, tRNA ligases, proteases and proteins associated to the *S*-layer were increased. The higher expression rates of these proteins might be controlled by specific histidine kinases and transcriptional regulator of FNR/CRP family, which appeared to be higher abundant as well. Many of these molecular processes require ATP for being active, which is produced in the TCA cycle. Throughout the early stages of repair, *D. radiodurans* needs a large quantity of ATP and uses its preferred carbon source, amino acids, as energy resource, which was indicated by the low quantity of extracted metabolites and TCA cycle intermediates from the vacuum treated cells. This study gives insights how *D. radiodurans* cope with the vacuum conditions on a molecular scale, but in addition, it shows interesting opportunities for future mutant-based studies, as important marker proteins are emphasized. As high vacuum causes severe damage to the cell wall, mutant studies with *S*-layer proteins, e.g., SlpA or DR_0774 together with the putative regulatory type III histidine kinases appears very attractive. A combination of shotgun proteomics with imaging techniques could help to define the roles of these proteins in restoring the cell wall after the vacuum exposure.

## Author Contributions

EO, YK, NÖ, and ER performed the experiments. All authors provided the editorial input, made substantial contributions to the acquisition, analysis, and interpretation of data described in this manuscript, and critically reviewed the report and approved the final version.

## Conflict of Interest Statement

The authors declare that the research was conducted in the absence of any commercial or financial relationships that could be construed as a potential conflict of interest.
