# Supplementary figures and images for: Proteomic and Metabolomic Profiling of Deinococcus radiodurans Recovering After Exposure to Simulated Low Earth Orbit Vacuum Conditions
Source: Front Microbiol. 2019 Apr 29;10:909. doi: 10.3389/fmicb.2019.00909 (PMC6501615; doi:10.3389/fmicb.2019.00909)

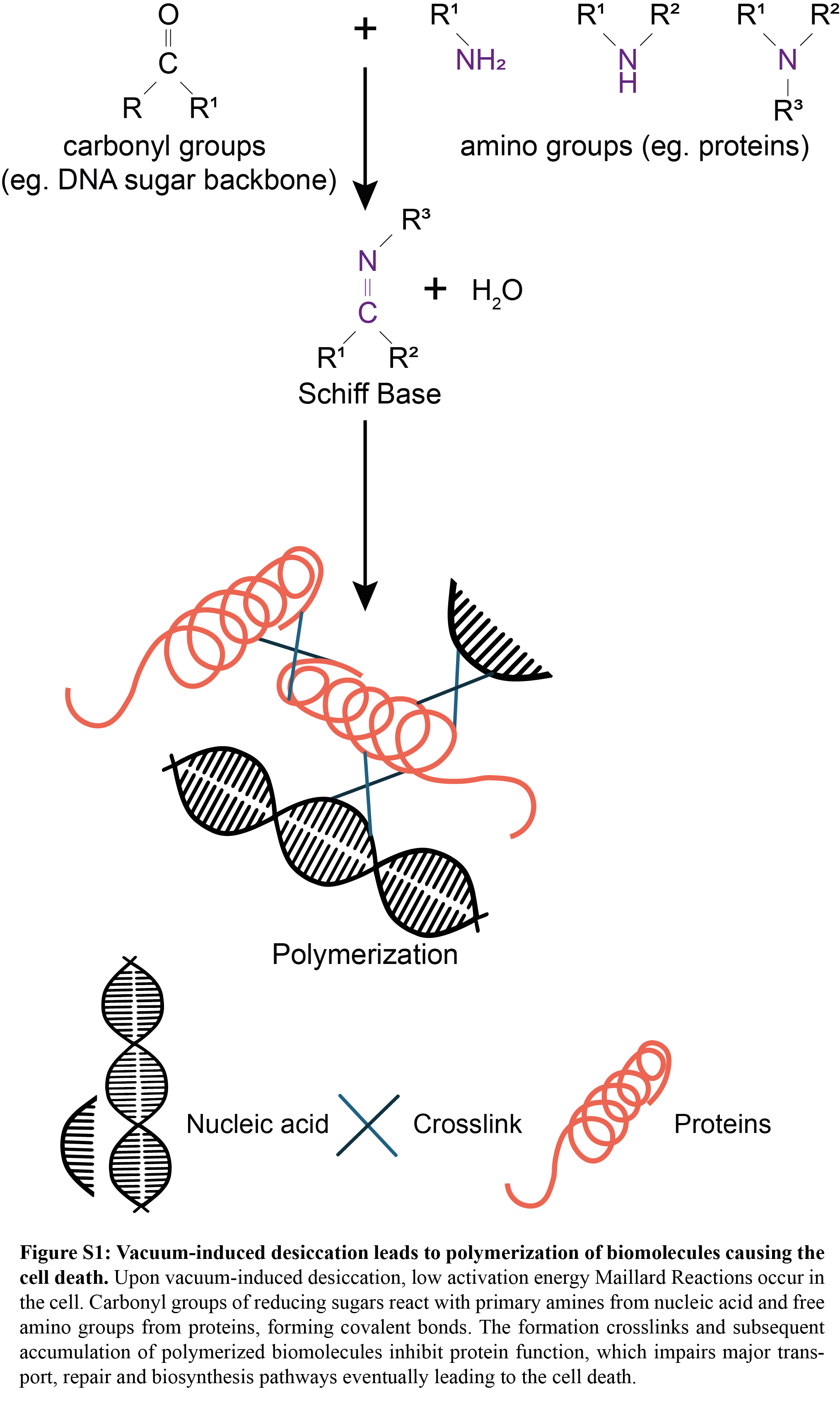

Supplement: Supplementary file 4 [file Image_1.JPEG]

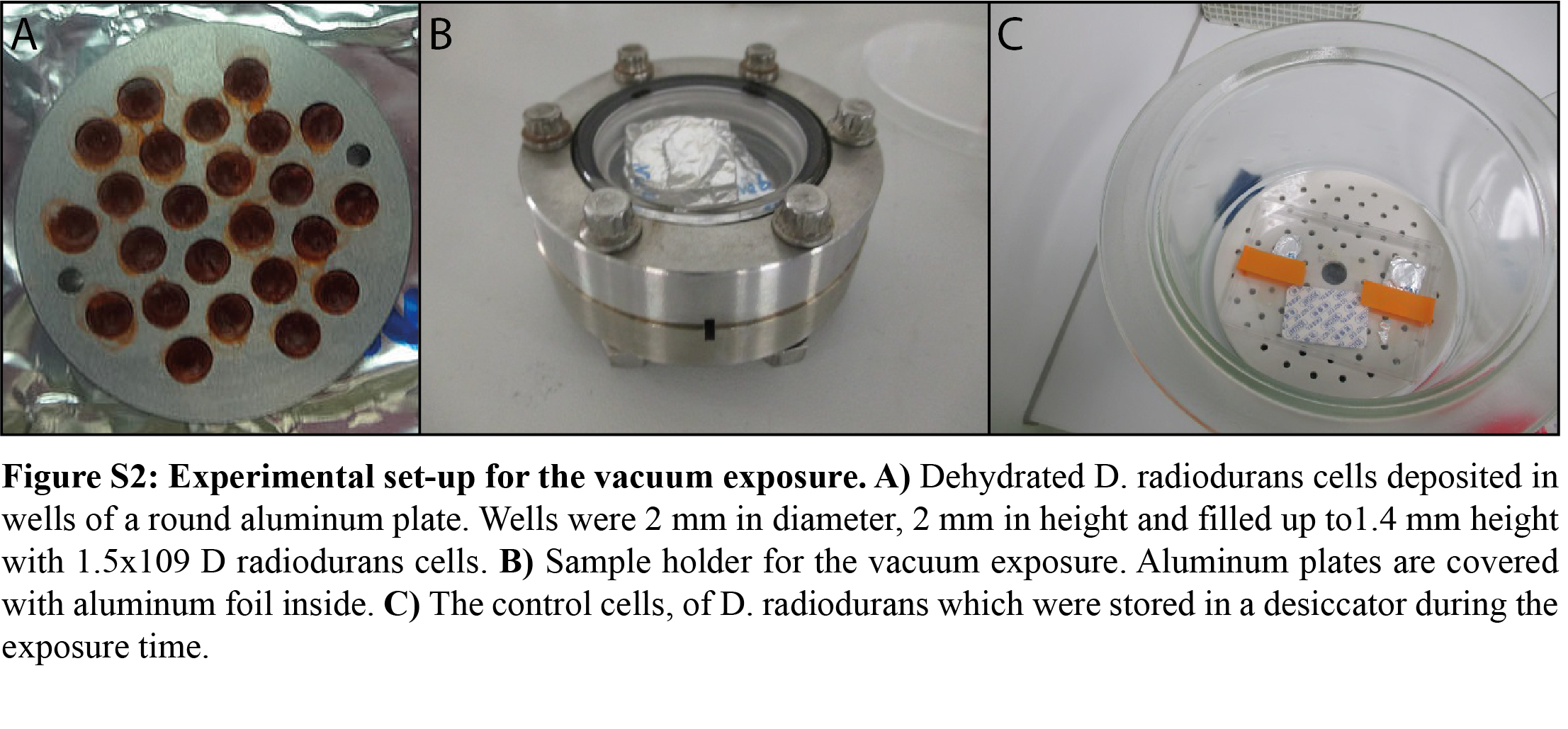

Supplement: Supplementary file 5 [file Image_2.JPEG]

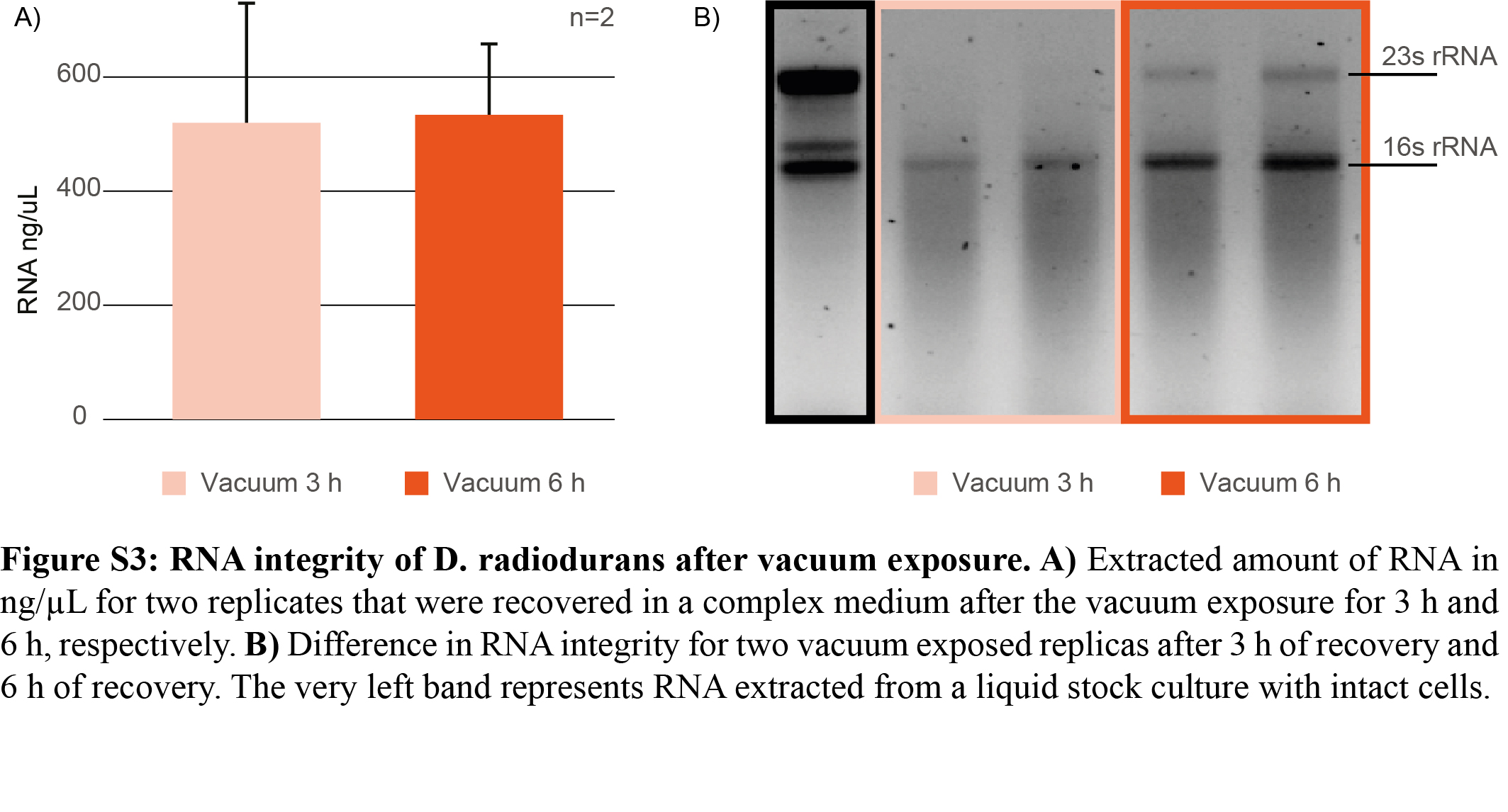

Supplement: Supplementary file 6 [file Image_3.JPEG]

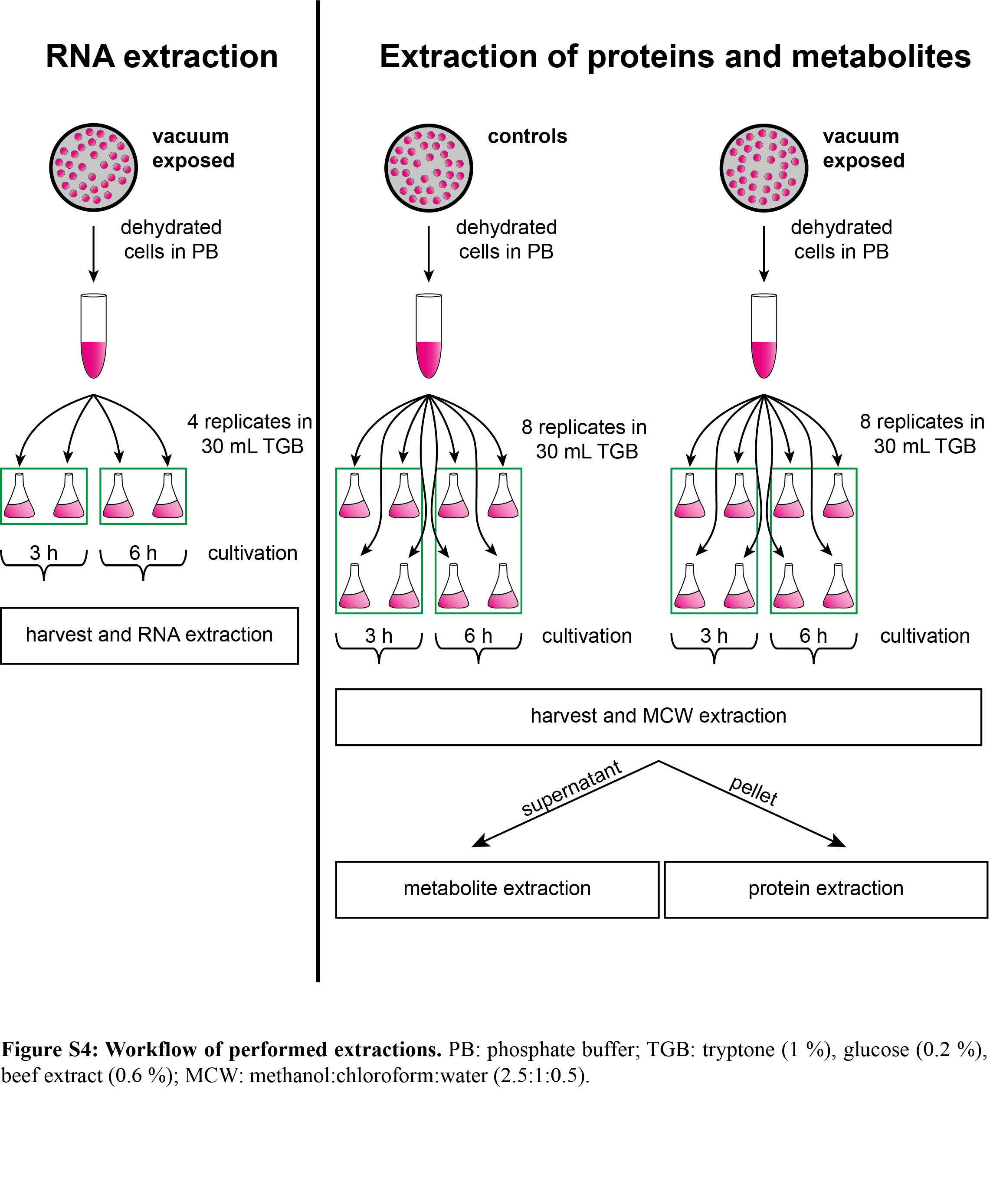

Supplement: Supplementary file 7 [file Image_4.JPEG]

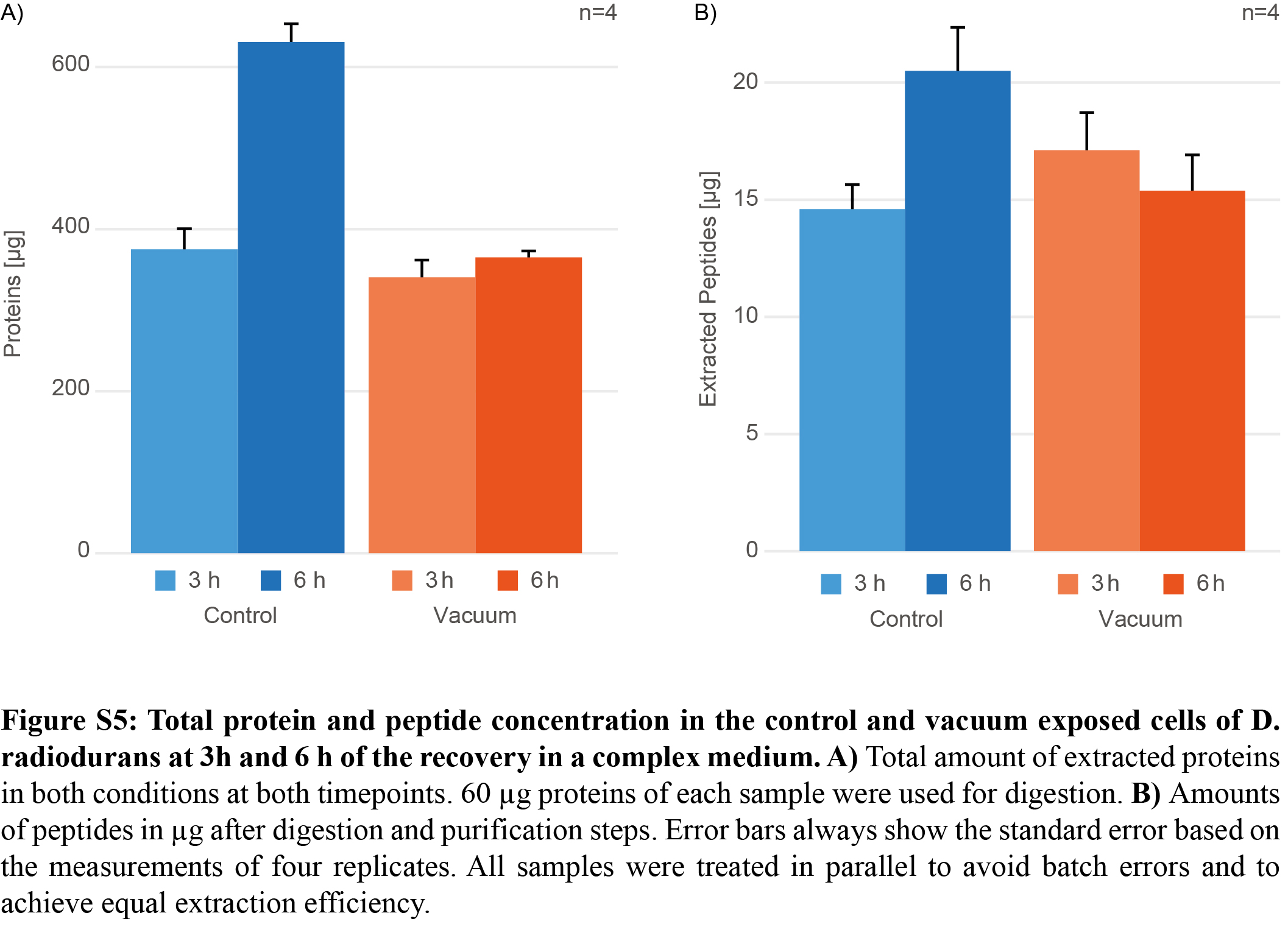

Supplement: Supplementary file 8 [file Image_5.JPEG]

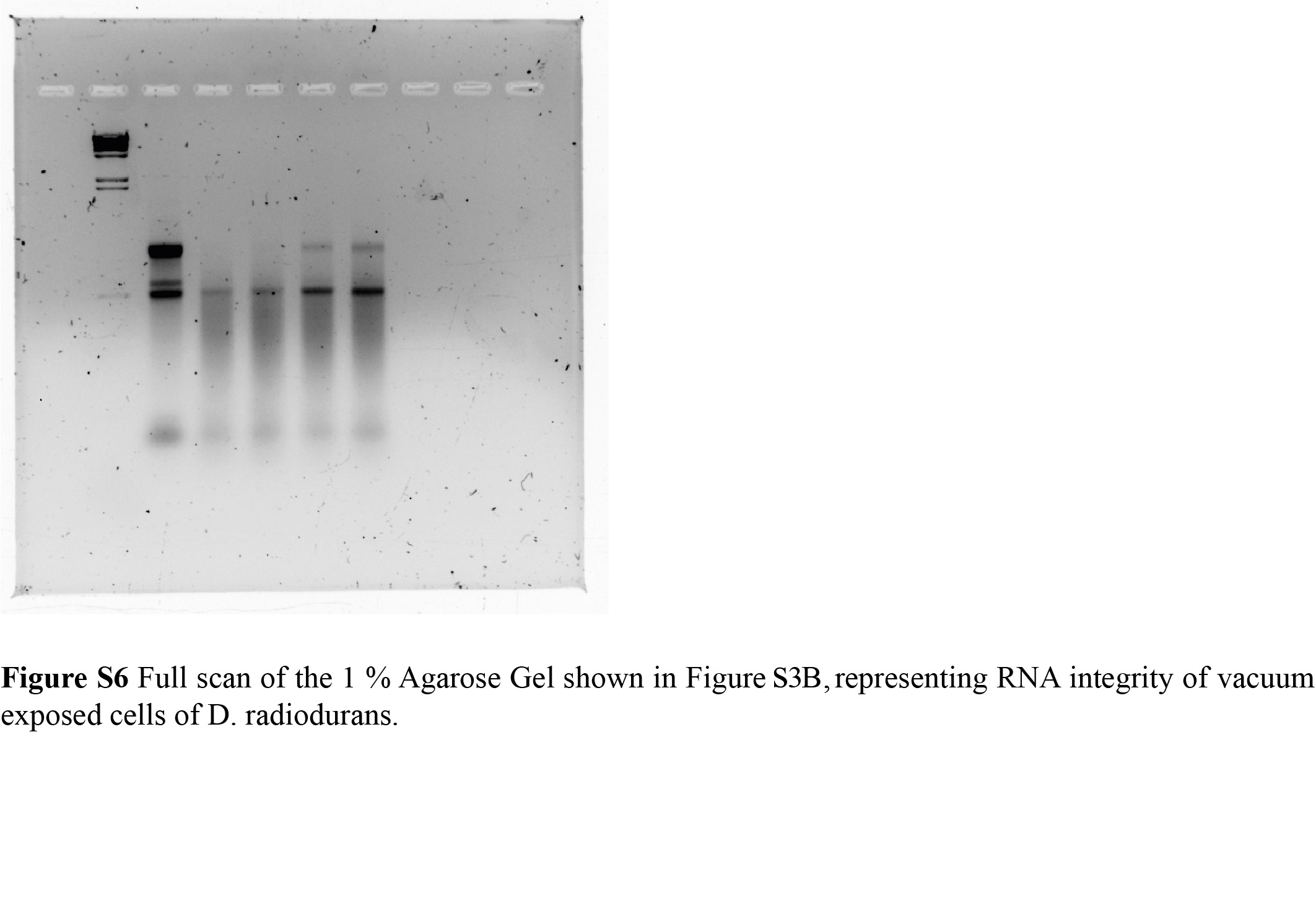

Supplement: Supplementary file 9 [file Image_6.JPEG]
